# Medical Students’ Perception of the Usage of Lecture Recording Software

**DOI:** 10.7759/cureus.2963

**Published:** 2018-07-11

**Authors:** Azhar Hussain, Elsa Tabrez, Amitabha Basu, Caron S M D'Silva

**Affiliations:** 1 Medicine, Xavier University School of Medicine, Oranjestad, ABW; 2 Medicine, St. Matthew’s University School of Medicine, George Town, CYM; 3 Dean of Basic Science and Professor of Pathology, St. Matthew's University School, George Town, CYM; 4 Associate Professor of Pharmacology, Biostatistics, and Epidemiology, St. Matthew's University, School of Medicine, George Town, CYM

**Keywords:** surveymonkey®, lecture recording software, statistical software

## Abstract

Background: Lecture recording software is a useful reference tool that allows students to revisit lectures and understand complicated concepts in higher education. It is also a useful tool for students with learning difficulties, allowing them to reference and learn the material at their own pace. A significant advantage of this tool is the accessibility of course material to the students off campus. This study attempted to learn the students’ perception of the purpose, use, and benefit of lecture recording software at a medical school.

Methods: The study was conducted using a structured questionnaire delivered, via an Internet-based survey application in the Fall semester of 2017, to 105 students attending the basic sciences courses. A web link was generated after the 18-point questionnaire was uploaded to an online survey software. The link was communicated electronically to each student along with the date and time of the survey. The survey was anonymous. The results of the survey were summarized using descriptive statistics and graphical methods. Students were asked to submit voluntary, informed consent to participate in the study before attempting to answer the questionnaire. The institutional review board approved the research.

Results: The results showed 77% students used this resource to understand points they missed in the class, 75% of them relearned complex ideas/concepts, and 62% of them used it to rewrite class notes. Reportedly, the software was used by students (78%) who missed a class due to an illness or while attending clinical shadowing. Of the students, 87% agreed that the software is helpful because of its off-campus availability while 84% of the students liked the service, as it allowed them to listen to the lectures at their own pace. Many students (65%) felt that the service helped them score better in the exams, whereas 38% did not think the recordings was helpful to get the desired grade and 50% student felt it was time-consuming.

Conclusion: Despite the time-consuming listening process, students expressed a positive opinion about the usefulness of this software. Recording and archiving class lectures could be a useful academic resource. Students could learn from these archived lectures before the class and engage in the discussion later, enhancing active learning. The result suggests that students should also use other study resources and methods to achieve the desired grades. The induction of this student service into a professional curriculum would enhance the students’ satisfaction, effectiveness, and outcomes.

## Introduction

Information delivery methods in higher education have changed considerably with the increased use of lecture recording software in recent years [1]. Lecture recordings are useful study tools and class reference material for students enrolled in on-campus or online courses. The video recording of lectures allows the student to follow the voice and notes, as the professor progresses through Microsoft PowerPoint (Microsoft Corporation, Redmond, Washington, US) presentations [2]. It is also a useful tool for students with learning difficulties because it allows them to reference and learn the material at their own pace. A significant advantage of this tool is the accessibility of course material to the students, wherever and whenever they need it [3]. Students attending St. Matthew’s University both on location during basic sciences and off location during clinical sciences have access to all recorded lectures. At St. Matthew’s University, lectures are recorded through the lecture recording software known as Panopto (Seattle, Washington, US) on the classroom computer; it records the voice, computer screen, and note-taking by the faculty. This research is the first attempt to understand the student’s perception of the lecture recording application available at the university.

The conceptual/theoretical framework

The use of information technology in education, the patterns of usage, and its benefit have been studied in recent times. It is presumed that these tools enhance the learning experience of students [3]. Several studies describe students’ affinity to the use of web-based learning [4-5] while other quasi-experimental studies show mixed results related to the effectiveness of recorded lectures [6-7]. This paper attempts to add to the growing body of literature regarding the use and perceived effectiveness of web-based tools for learning.

Students and faculty members at St. Matthew’s University have been using Panopto for five years. This research attempts to understand the medical students’ perception of this application. To achieve this purpose, a study method that was descriptive, measurable, objective, and interpreted as-is-described by students were considered. The Internet survey method was feasible to gather data from a large number of students, easy to deliver, economical, and it suited the university’s infrastructure. The results obtained from the survey have a generalizability that can be applied globally.

Research question

We assumed that the students have a definitive perception of the use of the lecture recording software and its benefits. Based on this ontological assumption, a three-point research questionnaire was formulated, which addressed the following:

1. The purpose of the use of the lecture recording application

2. The frequency of use of this lecture recording application by students

3. The students’ perception of the effectiveness of this application

## Materials and methods

Study design

This study is a cross-sectional, descriptive, questionnaire study using the Internet survey method. In this manuscript, search engines, such as PubMed, Google Scholar, and DiscoverEd, were used to obtain peer-reviewed articles to gain an understanding of the topic before developing a suitable questionnaire to answer the proposed research question.

Time period

The survey was conducted just before the first lecture of the day, during the eighth week of the Fall 2017 semester.

Population

Medical students of St. Matthew’s University's basic sciences and clinical sciences. The Internet survey was delivered in the form of a questionnaire [1-2]. The 18-point questionnaire was uploaded to an online survey software called SurveyMonkey (San Mateo, CA, US), and a web link was generated that was communicated electronically through Moodle (software used by St. Matthew’s University for official communication) to each student along with the date and time of the survey. The student’s personal information remained confidential and the students’ participation was completely voluntary. This survey contained a total of 18 questions in the three categories mentioned above in the research question section. The students responded with either yes/no answers for the first two categories of questions and, for the third category of questions, with one of the following options: strongly agree, agree, disagree, or strongly disagree.

Statistical analysis

The data was downloaded from SurveyMonkey in Microsoft Excel (Redmond, Washington, US) format and was analyzed using the appropriate statistical software. Results were reported in the form of descriptive statistics and graphical presentations.

Ethical aspect

The research was conducted only after receiving approval from the institutional review board/ethics committee on September 27, 2017. The information sheet and consent form were uploaded online to the SurveyMonkey software. The students gave voluntary, informed consent to participate in the study before attempting to answer the survey questionnaire. All personal information gathered from the students is strictly confidential.

## Results

**Figure 1:**
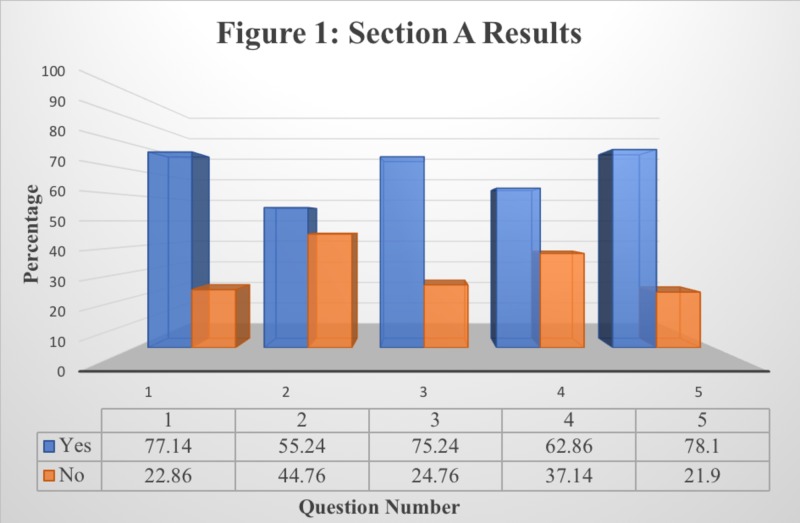
Responses to Section A: Purpose of Use of Lecture Recording Application

**Figure 2:**
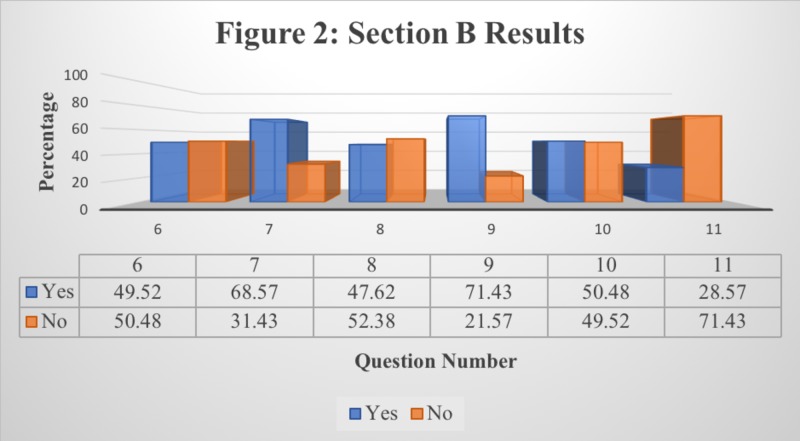
Responses to Section B: Students’ Usage Pattern of Lecture Recording Application

**Figure 3:**
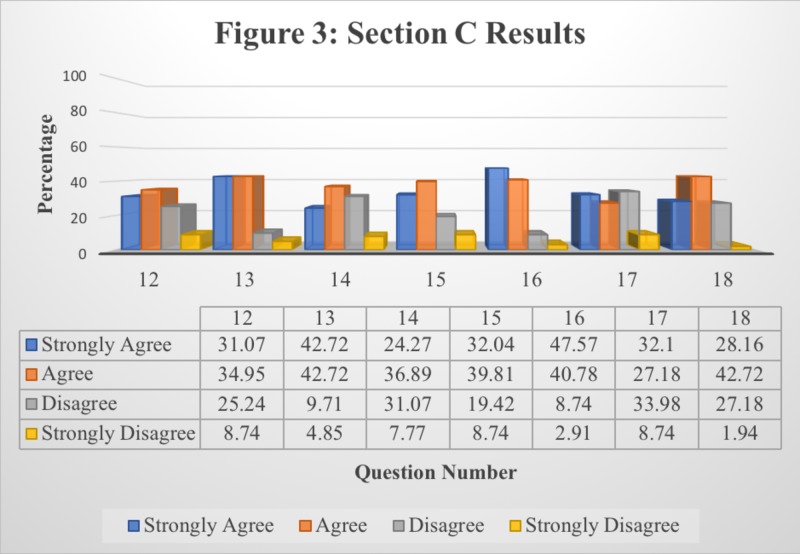
Responses to Section C: Students’ Overall Perception of Lecture Recording Application

The research conducted involved the responses of 103 individual students from St. Matthew’s University School of Medicine. Tables 1-3 display the results attained from the students’ responses to each question in each section provided. Figures 1-3 display the same results in a bar graph format. Results are recorded in terms of the percentage of the population’s response per question.

**Table 1:** Responses to Section A: Purpose of Use of Lecture Recording Application

	Yes	No
I use Panopto^TM^ (Lecture Recording Software) to pick up on points I missed.	77.14%	22.86%
I use Panopto^ TM^ (Lecture Recording Software) to pick up announcements and exam hints.	55.24%	44.76%
I use Panopto^TM^ (Lecture Recording Software) to revisit complex ideas and concepts when the lectures are unclear.	75.24%	24.76%
I use Panopto^ TM^ (Lecture Recording Software) to take comprehensive notes and or correct notes taken in the classroom.	62.86%	37.14%
I use Panopto^ TM^ (Lecture Recording Software) when I am on sick leave or shadowing with a clinical preceptor.	78.10%	21.90%

**Table 2:** Responses to Section B: Students’ Usage Pattern of Lecture Recording Application

	Yes	No
I use Panopto^ TM^ (Lecture Recording Software) to listen to the entire recording.	49.52%	50.48%
I choose particular segments of the recording while listening to Panopto^TM^ (Lecture Recording Software).	68.57%	31.43%
I use Panopto^TM^ (Lecture Recording Software) to listen to lecture notes regularly.	47.62%	52.38%
I have used Panopto^ TM^ (Lecture Recording Software) to browse and stop at points of interest.	71.43%	28.57%
I have used Panopto^ TM^ (Lecture Recording Software) to listen to multiple lectures a day.	50.48%	49.52%
I use Panopto^ TM ^(Lecture Recording Software) infrequently and only use it before the examination.	28.57%	71.43%

**Table 3:** Responses to Section C: Students’ Overall Perception of Lecture Recording Application

	Strongly agree	Agree	Disagree	Strongly disagree
Panopto^TM^ (Lecture Recording Software) helped me to pass the course and/or block exam.	31.07%	34.95%	25.24%	8.74%
By using Panopto^ TM^ (Lecture Recording Software), I can work at my own pace.	42.72%	42.72%	9.71%	4.85%
Panopto^TM^ (Lecture Recording Software) helped me to achieve my desired scores.	24.27%	36.89%	31.07%	7.77%
I find that Panopto^TM^ (Lecture Recording Software) helped me in preparing for exams effectively.	32.04%	39.81%	19.42%	8.74%
I can view the recorded lectures anywhere (outside the campus), anytime through Panopto^TM^ (Lecture Recording Software).	47.57%	40.78%	8.74%	2.91%
It is too time-consuming to relisten to the lectures on Panopto^TM^ (Lecture Recording Software).	32.10%	27.18%	33.98%	8.74%`
Panopto^TM^ (Lecture Recording Software) is usable on all types of electronic devices including cell phones, iPads, laptop computers, etc...	28.16%	42.72%	27.18%	1.94%

## Discussion

Table 1 presents the data collected from Section A of the survey questionnaire while Figure 1 presents a graphical interpretation for a better understanding. This section was designed to determine the purpose of using the lecture recording application from the perspective of the students. According to the results attained, a majority of the student population utilizes Panopto (Seattle, Washington, US) to pick up on missed lecture points, revisit complex concepts, compile notes, and listen to the lecture if they did not attend in person. While a larger portion of the student population agrees upon the importance of Panopto in terms of its purpose and usage, Question 2 suggests that almost half of the student population uses Panopto to recall announcements (55.24%) while the other half does not (44.76%). These results indicate that the majority of St. Matthew’s University School of Medicine students’ avail Panopto as a lecture recording software and recognize its purpose.

Table 2 presents the data collected from Section B of the survey questionnaire while Figure 2 presents a graphical interpretation for better understanding. This section was designed to determine the frequency of use of Panopto as a lecture recording application as well as the students’ usage pattern of the software. It can be deduced that a predominant portion of the student population chooses specific segments of a recording while using Panopto as per the data collected in Questions 7 and 9. The results attained in Question 11 suggest that a majority of students (71.43%) use Panopto frequently, not just prior to an examination. According to Questions 6, 8, and 10, about roughly half of the student population listens to the entire recording, listens to the recordings regularly, and listens to multiple lectures in a day while the other half does not.

Table 3 presents the data collected from Section C of the survey questionnaire, while Figure 3 presents a graphical interpretation for better understanding. This section was designed to determine the students’ perspective of the effectiveness of Panopto as a lecture recording application. The results in this section were obtained by the student choosing either “Strongly Agree,” “Agree,” “Disagree,” or “Strongly Disagree.” Data collection in the form of a Likert scale was used to assess the responses in terms of generating a range as well as allowing the student to choose an answer that best aligns with their views as opposed to a simple yes or no. This form of data collection allows a better understanding of the students’ opinions regarding how effective the lecture recording application is.

According to the data collected in Table 3, a majority of the student population is in favor of the positive, effective benefits of Panopto. This conclusion can be made while noting the results collected from Questions 12, 13, 15, and 16, where a large portion of the student population chose either “Strongly Agree” or “Agree” in concordance with the effectiveness of Panopto. As a result, it can be concluded that Panopto allows students to work at their own pace, access Panopto in their own time, as well as prepare for and pass block exams. However, in Question 14, even though a larger number of the student population chose options in favor of Panopto, helping them achieve their desired scores, the percentage of students who “Disagree” on the same matter is relatively high (31.07%). Results obtained in Question 17 suggest that it is too time-consuming to relisten to Panopto recordings; this conclusion can be drawn by noting the highest percentage in accordance with the “Disagree” option (33.98%).

## Conclusions

The results acquired by the 18-question student survey demonstrates overall student satisfaction with Panopto, the lecture recording application, with regards to its purpose, the frequency of use, and effectiveness. While some data can be considered inconclusive owing to a small percentage difference between overall student opinions, a majority of the data is suggestive of students having a positive perception of the software. When considering the opposite, the collected data also indicates that students perceive relistening to recordings to be too time-consuming. Data also alludes to the fact that the majority of students do not feel that Panopto has helped them achieve their “desired” scores. However, it needs to be taken into consideration that a larger population of students believed that Panopto helped them pass the course and/or block exams. These two data points can suggest that while students are passing their exams, they are not necessarily achieving their desired scores.
